# *Epilepsia open*—April 2026 announcements

**DOI:** 10.1002/epi4.70254

**Published:** 2026-04-03

## ILAE CONGRESSES

Curso virtual: Correlación anátomo‐electro‐clínica

March 22– May 2, 2026

Online

5th International Summer School of Neuropsychology

April 19–24, 2026

Picardy, France

8th East Mediterranean Epilepsy Congress

April 30– May 2, 2026

Al Khobar, Saudi Arabia

ILAE School on Neuroimaging 2026

May 6–9, 2026

Potsdam, Germany

XIV Congreso Latinoamericano de Epilepsia

May 16–18, 2026

Lima, Peru

ILAE Summer School on EEG and Epilepsy

July 11–19, 2026

Dianalund, Denmark & Online

1° Curso Latinoamericano de EEG Pediátrico

August 6–9, 2026

TBC

16th European Epilepsy Congress.

September 5–9, 2026

Athens, Greece

16th International Summer School for Neuropathology and Epilepsy Surgery

September 10–13, 2026

Erlangen, Germany

16th Asian & Oceanian Epilepsy Congress

November 19–22, 2026

Kuala Lumpur, Malaysia

## 2027

16th ILAE School on Pre‐Surgical Evaluation for Epilepsy and Epilepsy Surgery

January 15, 2027

Brno, Czech Republic

37th International Epilepsy Congress

August 28—September 1, 2027

Amsterdam, Netherlands

## ILAE WEBINARS

ILAE‐Africa 7 April Webinar

April 7, 2026

Posterior Quadrant Epilepsy

April 10, 2026

Épilepsies de l’adolescent

April 14, 2026

Definitions – how do they work in epilepsy (ILAE e‐Forum Series 2026)

April 15, 2026

ILAE‐Africa 21 avril webinaire

April 21, 2026

Created Just for You: The ILAE Epilepsy Nursing Curriculum

April 22, 2026

ILAE‐EMR 24 April webinar

April 24, 2026

## OTHER CONGRESSES

EPIPED Course: Treatment Strategies in Pediatric Epilepsies

April 22–25, 2026

Girona, Spain

18th Eilat Conference on New Antiepileptic Drugs and Devices

May 3–6, 2026

Madrid, Spain

International Conference for Post‐Traumatic Epilepsy 2026

May 3–6, 2026

Princeton, New Jersey, USA

Paros Epilepsy 2026

May 11–15, 2026

Paros, Greece

20th Specialist Epilepsy Teaching Weekend

May 16–17, 2026

Birmingham, UK

13th International Residential Course on Drug Resistant Epilepsies

May 17–23, 2026

Tagliacozzo, Italy

64th Annual Meeting of the German Society for Epileptology

June 10–13, 2026

Würzburg, Germany

Paediatric Epilepsy Training 2—Leeds

June 11–12, 2026

Leeds, UK

Paediatric Epilepsy Training 3—Leeds

June 11–12, 2026

Leeds, UK

22nd San Servolo Advanced Epilepsy Course

July 20–31, 2026

San Servolo (Venice), Italy

Epilepsy Imaging in Resource Strained Settings

August 31–September 12, 2026

TBC

Workshop on Combined EEG‐fMRI in epilepsy

September 9–10, 2026

Athens, Greece

2026 CLAE Annual Scientific Meeting

October 2–4, 2026

Niagara‐on‐the‐Lake, Ontario, Canada

43rd International Conference on Advances in Psychiatry and Mental Health

October 5–6, 2026

Dubai, United Arab Emirates

ILAE British Branch 2026 Annual Scientific Conference

October 8–10, 2026

Harrogate, UK

Annual Meeting of the German‐Austrian‐Swiss Epilepsy Working Groups 2026

October 27–30, 2026

Vienna, Austria

1st Swiss Neuro Week

November 18–20, 2026

Bern, Switzerland

Encephalitis 2026

December 7–8, 2026

London, UK & Online

